# Supplementary material for: Mechanisms governing the pioneering and redistribution capabilities of the non-classical pioneer PU.1
Source: Nat Commun. 2020 Jan 21;11:402. doi: 10.1038/s41467-019-13960-2 (PMC6972792; doi:10.1038/s41467-019-13960-2)
Supplement: Supplementary file 7 — Source data [file 41467_2019_13960_MOESM7_ESM.zip › Source_Data/FigureS2/FigureS2B_MotifScanOutput/KmeansCluster10/homerResults/motif8.similar.html]

motif8

## Information for motif8

G
C
T
A
C
G
T
A
T
C
G
A
A
G
T
C
A
G
T
C
C
G
T
A
T
A
G
C
G
C
T
A
C
G
A
T
  
Reverse Opposite:  

C
G
T
A
C
G
A
T
A
T
C
G
A
C
G
T
A
C
T
G
A
C
T
G
A
G
C
T
G
C
A
T
C
G
A
T
  

|  |  |
| --- | --- |
| p-value: | 1e-89 |
| log p-value: | -2.066e+02 |
| Information Content per bp: | 1.682 |
| Number of Target Sequences with motif | 889.0 |
| Percentage of Target Sequences with motif | 23.95% |
| Number of Background Sequences with motif | 5080.9 |
| Percentage of Background Sequences with motif | 11.50% |
| Average Position of motif in Targets | 100.3 +/- 52.8bp |
| Average Position of motif in Background | 100.2 +/- 64.3bp |
| Strand Bias (log2 ratio + to - strand density) | 0.1 |
| Multiplicity (# of sites on avg that occur together) | 1.13 |
| Motif File: | file (matrix) reverse opposite |

### Similar de novo motifs found

|  |  |  |  |  |  |  |  |
| --- | --- | --- | --- | --- | --- | --- | --- |
| Rank | Match Score | Redundant Motif | P-value | log P-value | % of Targets | % of Background | Motif file |
| 1 | 0.976 | G A C T T C G A T C G A A T G C A G T C C G T A A T G C G T C A | 1e-88 | -203.118804 | 34.78% | 20.03% | motif file (matrix) |
| 2 | 0.985 | A G T C G C T A C T G A T G C A G A T C A T G C C T G A T A G C G T C A C G T A | 1e-77 | -179.238212 | 19.15% | 8.73% | motif file (matrix) |
| 3 | 0.972 | A C G T A T C G A C G T A C T G A C T G A C G T A G C T C G A T A T C G G T A C A G T C | 1e-67 | -154.774109 | 21.47% | 11.03% | motif file (matrix) |
| 4 | 0.959 | C G T A C A G T A C G T G A C T A C G T A C T G A C G T A C T G A T C G A C G T A G C T C G A T | 1e-61 | -142.737814 | 13.44% | 5.66% | motif file (matrix) |
| 5 | 0.950 | A C G T A C T G A G C T A C T G A C T G A C G T A C G T | 1e-48 | -112.051256 | 15.09% | 7.54% | motif file (matrix) |
| 6 | 0.845 | T G C A T G C A G T A C A G T C C G T A G T A C G T C A C G A T T G A C G C T A T C G A C G T A T A C G | 1e-39 | -89.879440 | 6.63% | 2.39% | motif file (matrix) |
| 7 | 0.807 | G C T A T G A C G C A T A G C T A C G T A C T G A C G T T C A G T C A G A G C T A G C T G A C T G A T C | 1e-17 | -41.107919 | 1.54% | 0.32% | motif file (matrix) |
| 8 | 0.757 | C G T A C G T A A G T C A G T C C T G A A T G C A T G C | 1e-13 | -30.179021 | 11.26% | 7.66% | motif file (matrix) |
